# Supplementary material for: Cre-Controlled CRISPR mutagenesis provides fast and easy conditional gene inactivation in zebrafish
Source: Nat Commun. 2021 Feb 18;12:1125. doi: 10.1038/s41467-021-21427-6 (PMC7893016; doi:10.1038/s41467-021-21427-6)
Supplement: Supplementary file 1 — Supplementary Information [file 41467_2021_21427_MOESM1_ESM.pdf]

Supplementary table 1: 3C *tyr* with and without Cre mRNA injections

No FP				
**#**	**area**	**mean**	**min**	**max**
**1**	2.048	4.185	0	45
**2**	2.048	5.037	0	37
**3**	2.048	6.614	0	31
**4**	2.048	5.392	0	32
**5**	2.048	4.979	0	19
**6**	2.048	5.384	0	25
**7**	2.048	3.692	0	9
**8**	2.048	4.198	0	12
**9**	2.048	8.144	0	35
**10**	2.048	5.831	0	21
**11**	2.048	3.720	0	12
**12**	2.048	3.771	0	9

DsRed+				
**#**	**area**	**mean**	**min**	**max**
**1**	2.048	4.914	0	16
**2**	2.048	5.543	0	24
**3**	2.048	6.584	0	26
**4**	2.048	4.362	0	12
**5**	2.048	5.208	0	23
**6**	2.048	7.328	0	22
**7**	2.048	5.047	0	21
**8**	2.048	4.939	0	19
**9**	2.048	5.573	0	16
**10**	2.048	3.927	0	25
**11**	2.048	4.893	0	39
**12**	2.048	5.765	0	18

$tyr^{-/-}$				
**#**	**area**	**mean**	**min**	**max**
**1**	2.048	103.879	21	143
**2**	2.048	101.282	21	143
**3**	2.048	117.657	39	155
**4**	2.048	113.128	48	149
**5**	2.048	99.723	35	143
**6**	2.048	116.289	48	149
**7**	2.048	108.243	37	147
**8**	2.048	117.250	38	152
**9**	2.048	82.498	1	128
**10**	2.048	93.248	13	136
**11**	2.048	117.846	36	159
**12**	2.048	125.396	58	157

GFP+				
**#**	**area**	**mean**	**min**	**max**
**1**	2.048	83.726	1	144
**2**	2.048	79.802	0	146
**3**	2.048	121.518	40	158
**4**	2.048	114.247	26	159
**5**	2.048	114.600	0	163
**6**	2.048	112.647	8	161
**7**	2.048	108.337	0	159
**8**	2.048	91.584	14	139
**9**	2.048	108.566	37	162
**10**	2.048	89.157	17	144
**11**	2.048	104.769	21	142
**12**	2.048	101.873	41	147
**13**	2.048	88.893	14	156
**14**	2.048	80.115	7	132
**15**	2.048	87.852	1	152
**16**	2.048	86.030	1	143
**17**	2.048	106.040	0	162
**18**	2.048	75.653	7	129

Supplementary table 2: 3C *tyr* with and without *Tg(otx2b:CreER^{T2})*

No FP				
**#**	**area**	**mean**	**min**	**max**
**1**	2.048	9.565	0	62
**2**	2.048	10.573	0	60
**3**	2.048	11.958	0	63
**4**	2.048	6.884	0	37
**5**	2.048	6.870	0	22
**6**	2.048	7.542	0	27
**7**	2.048	5.571	0	27
**8**	2.048	9.364	0	33
**9**	2.048	5.652	0	19
**10**	2.048	4.992	0	20
**11**	2.048	6.885	0	35
**12**	2.048	6.360	0	26

DsRed+				
**#**	**area**	**mean**	**min**	**max**
**1**	2.048	8.210	0	25
**2**	2.048	8.022	0	41
**3**	2.048	6.807	0	50
**4**	2.048	10.189	0	31
**5**	2.048	10.279	0	38
**6**	2.048	6.541	0	58
**7**	2.048	10.359	0	28
**8**	2.048	7.439	0	28
**9**	2.048	10.000	0	37
**10**	2.048	10.759	0	37
**11**	2.048	7.827	0	22
**12**	2.048	10.211	0	40

tyr$^{-/-}$				
**#**	**area**	**mean**	**min**	**max**
**1**	2.048	101.551	24	139
**2**	2.048	102.992	40	140
**3**	2.048	106.451	25	137
**4**	2.048	110.719	25	149
**5**	2.048	115.108	36	153
**6**	2.048	96.699	16	135
**7**	2.048	108.866	29	143
**8**	2.048	101.146	24	142
**9**	2.048	107.771	32	141
**10**	2.048	104.236	45	145
**11**	2.048	109.805	33	145
**12**	2.048	89.508	10	123

GFP+				
**#**	**area**	**mean**	**min**	**max**
**1**	2.048	87.273	11	165
**2**	2.048	70.225	6	158
**3**	2.048	80.300	3	143
**4**	2.048	92.318	9	165
**5**	2.048	73.700	4	129
**6**	2.048	75.885	3	135
**7**	2.048	76.969	3	160
**8**	2.048	72.294	4	152
**9**	2.048	72.446	10	132
**10**	2.048	73.771	6	140
**11**	2.048	83.867	14	153
**12**	2.048	77.048	0	136
**13**	2.048	101.363	8	173
**14**	2.048	98.588	9	168
**15**	2.048	79.349	7	140
**16**	2.048	87.741	10	162
**17**	2.048	86.713	2	167
**18**	2.048	86.787	20	166

Supplementary table 3: primer list

GFP-for 5′-TATAACCGGTGAGATCTCCTAAGAAGAAGAGAAAGGTGGTGAGCAAGGGCGAGGAGC-3′

GFP-rev 5′-TATATCTAGACTCGAGGATCCGCTAGCGATACATTGATGAGTTT-3

U6a-for 5′- ATATGGTACCGGCGCGCCATATATCCCGGGGCGTCTTTTGTTCTGGTCATC-3′

U6a-rev 5′-ATATGAGCTCATGCTAGCAAAAAAGCACCGACTCGGTGCC-3′

tyr-for 5’-GGACTGGAGGACTTCTGGGGGT-3’

tyr-rev 5’-CCCCAGAAGTCCTCCAGTCCGA-3’

hsp70l-for 5’-CCGCAGAGAAACTCAACCGAAGAGAAGC-3’

Cas9-rev 5’-GTTTCCAAAGATAGGATGGCGCTCG-3’

tyr-P5-tail 5’-CACTCTTTCCCTACACGACGCTCTTCCGATCTGCCGGGCCAGACTGGACAGC-3’

tyr-P7-tail 5’-GTGACTGGAGTTCAGACGTGTGCTCTTCCGATCTGCCTCTCACTCTCCTCGACTCTTC-3’

tyr-off-P5-tail 5’-ACACTCTTTCCCTACACGACGCTCTTCCGATCTGGCAGACGGGTGAATTAGTGATGC-3’

tyr-off-P7-tail 5’- GTGACTGGAGTTCAGACGTGTGCTCTTCCGATCTAGCCAAGACTTGAACCAGCGAGC-3’

Supplementary figures

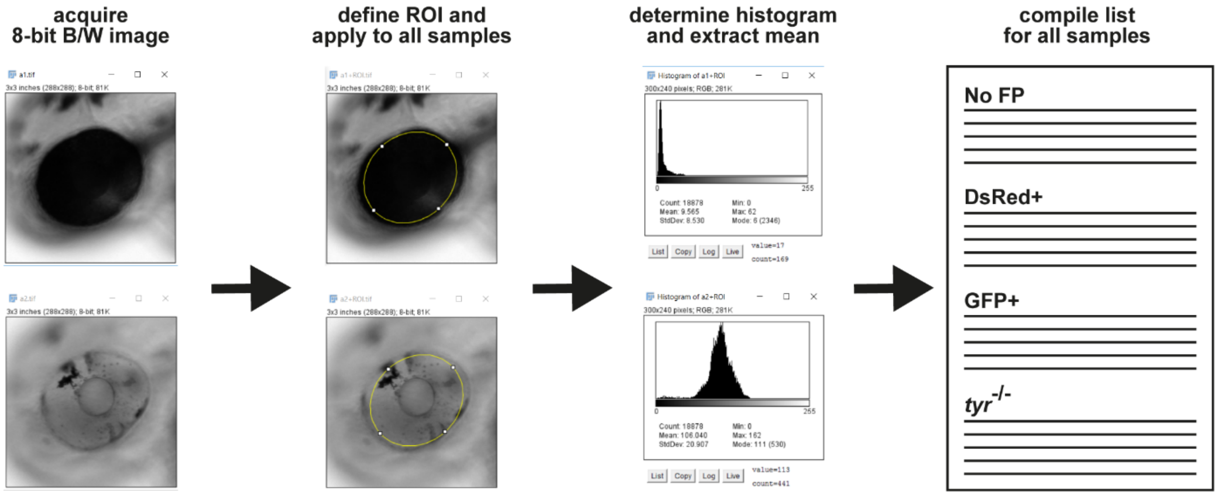

Supplementary figure 1: Schematic illustration of the quantification of the pigmentation in the retinal pigment epithelium. Following the acquisition of 8 bit black and white (B/W) images at 50 hpf, a region of interest (ROI, yellow ellipse), covering the dimension of the developing eye, was defined using the ROI manager of FIJI (ImageJ) and applied to all samples manually. Subsequently, the histogram was determined and the mean value was extracted. Finally, a list was compiled with the values for all images of embryos expressing no fluorescent protein (No FP), DsRed- (DsRed+) and GFP-positive (GFP+) siblings as well as *tyrosinase* mutant embryos (*tyr*$^{-/-}$).

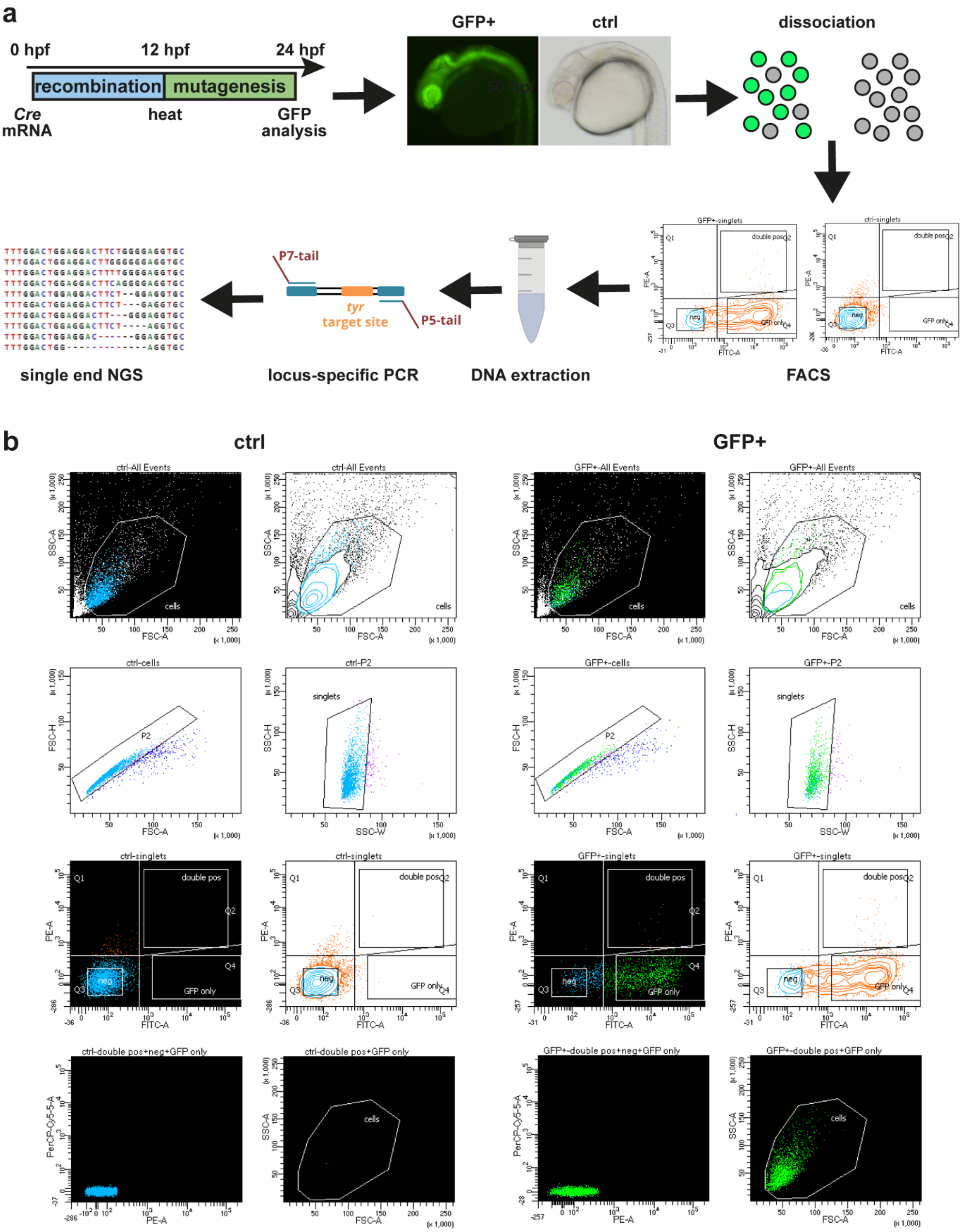

Tube: ctrl

Population	#Events	%Parent	%Total
All Events	18,169	####	100.0
cells	7,991	44.0	44.0
P2	7,024	87.9	38.7
singlets	6,713	95.6	36.9
Q1	145	2.2	0.8
Q2	3	0.0	0.0
double pos	2	66.7	0.0
Q3	6,541	97.4	36.0
neg	5,505	84.2	30.3
Q4	24	0.4	0.1
GFP only	1	4.2	0.0

Tube: GFP+

Population	#Events	%Parent	%Total
All Events	10,902	####	100.0
cells	4,127	37.9	37.9
P2	3,393	82.2	31.1
singlets	3,219	94.9	29.5
Q1	2	0.1	0.0
Q2	43	1.3	0.4
double pos	37	86.0	0.3
Q3	815	25.3	7.5
neg	411	50.4	3.8
Q4	2,359	73.3	21.6
GFP only	1,980	83.9	18.2

Supplementary figure 2: a Schematic illustration of experimental procedures. Following Cre mRNA injection and a heat treatment at 12 hpf, 30 GFP-positive embryos (GFP+) and 30 control siblings (ctrl) were collected at 24 hpf and dissociated prior to fluorescence-activated cell sorting (FACS). Upon genomic DNA extraction, the DNA was used as a template for a locus-specific PCR amplifying the *tyrosinase* (*tyr*) target region and the amplified PCR fragments were used in single-end next generation sequencing (NGS). **b** FACS sequential gating/sorting strategy of control and GFP-positive cells and their respective events during gating.

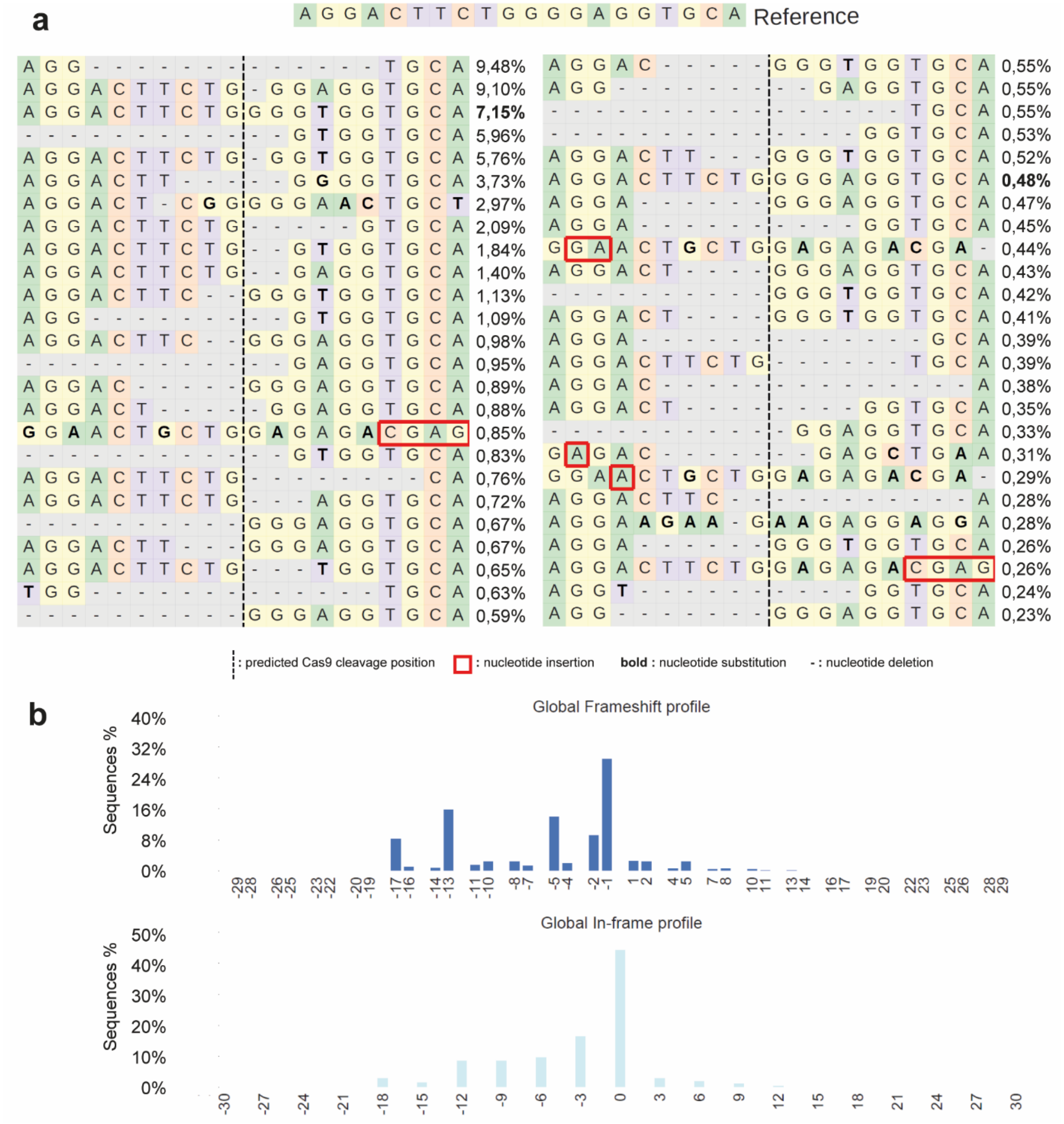

Supplementary figure 3: a Allele frequency table for GFP-positive cells. Distribution of identified alleles around the cleavage site for the guide GGACTGGAGGACTTCTGGGG. Nucleotides are indicated by unique colors (A = green; C = red; G = yellow; T = purple). Substitutions are shown in bold font. Red rectangles highlight inserted sequences. Horizontal dashed lines indicate deleted sequences. The vertical dashed line indicates the predicted cleavage site. **b** Profiles of frameshift and in-frame mutations given in percent.

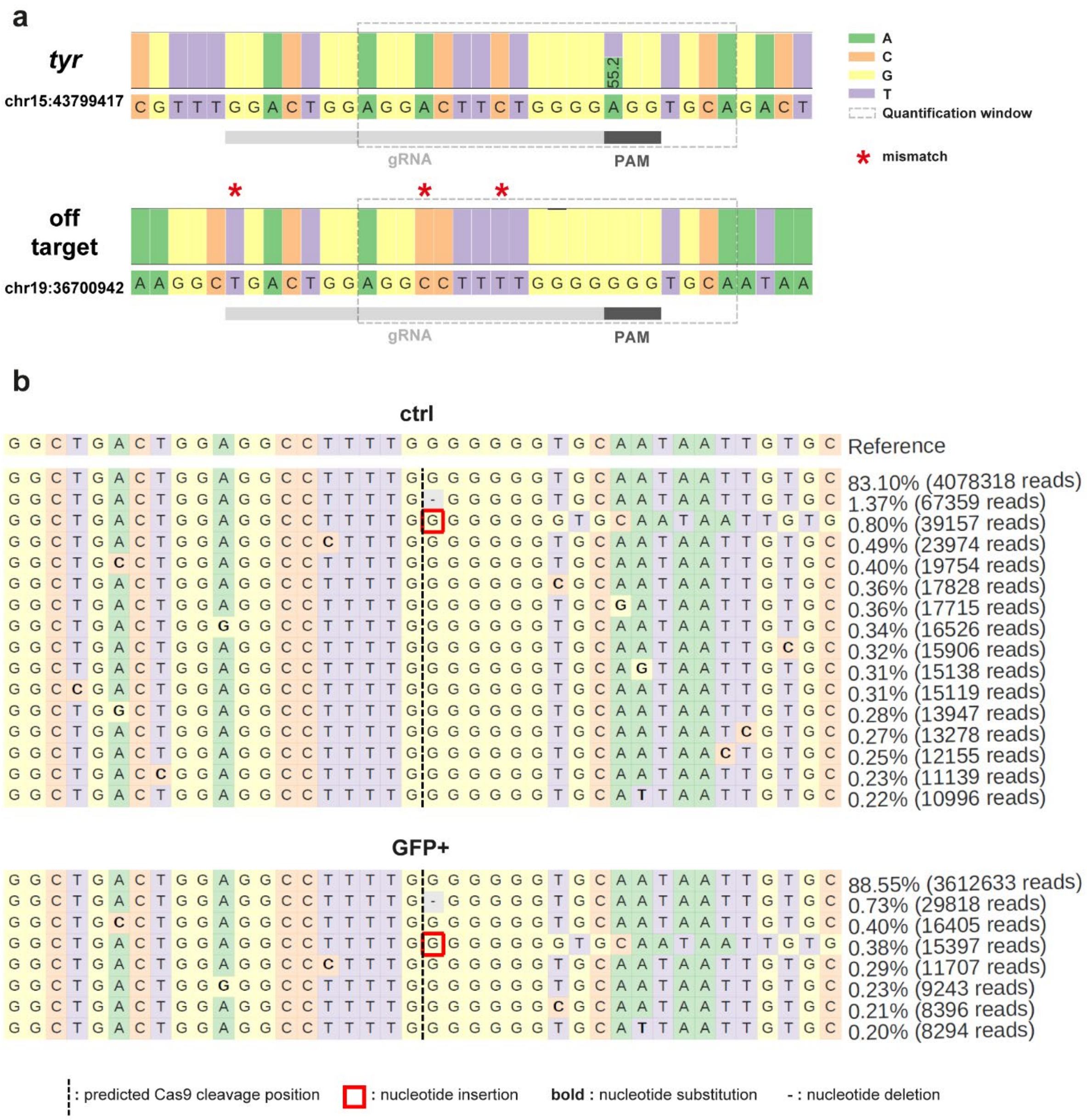

Supplementary figure 4: Off-target analysis. **a** The employed *tyr* gRNA target on chromosome 15 has a potential off-target on chromosome 19 with three mismatches (red asterisks). **b** Locus-specific sequencing reveals allele frequencies with 83.1% in control and 88.55% in GFP-positive cells. Nucleotides are indicated by unique colors (A = green; C = red; G = yellow; T = purple). Substitutions are shown in bold font. Red rectangles highlight inserted sequences. Horizontal dashed lines indicate deleted sequences. The vertical dashed line indicates the predicted cleavage site.

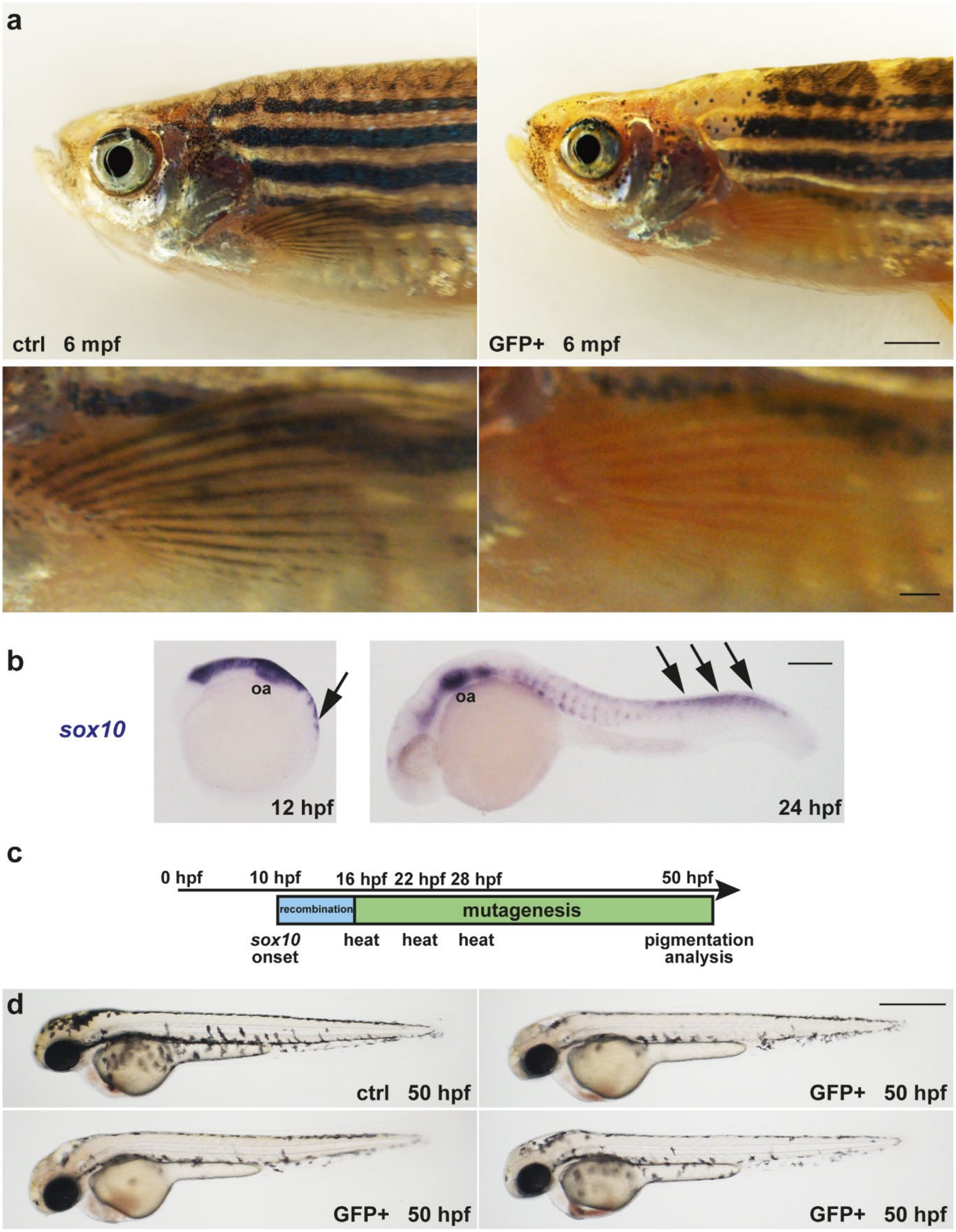

Supplementary figure 5: Pigmentation defects observed after *sox10*:Cre-mediated recombination. **a** Animals heat treated at 16 hpf show a significant loss of pigmentation at adulthood within the body and the pectoral fins. Examples shown are representative of eight individual fish examined. Scale bars: 2 mm and 500 µm for upper and lower row, respectively. **b** Expression of *sox10* shown by *in situ* hybridization at 12 and 24 hpf. Lateral views with anterior to the left. Arrows indicate expression of *sox10* in the posterior neural crest. Expression of *sox10* in the otic anlage (oa) serves as an additional landmark. Examples shown are representatives

across two experiments with >20 embryos showing the same result. Scale bar: 150 µm. **c** Timeline. Cre activity in *sox10*:Cre-positive animals at 10 hpf elicits recombination (blue box) in developing neural crest cells. Three consecutive heat treatments at 16, 22 and 28 hpf trigger expression of Cas9-GFP and the subsequent mutagenesis of the *tyr* target site (green box). Analysis of pigmentation was conducted at 50 hpf. **d** Further reduction in body pigmentation in triple heat treated animals. Examples shown are representatives across three experiments showing the same result. A total of >60 GFP-positive individuals and their respective non-GFP-positive siblings were analyzed. Scale bar: 500 µm.

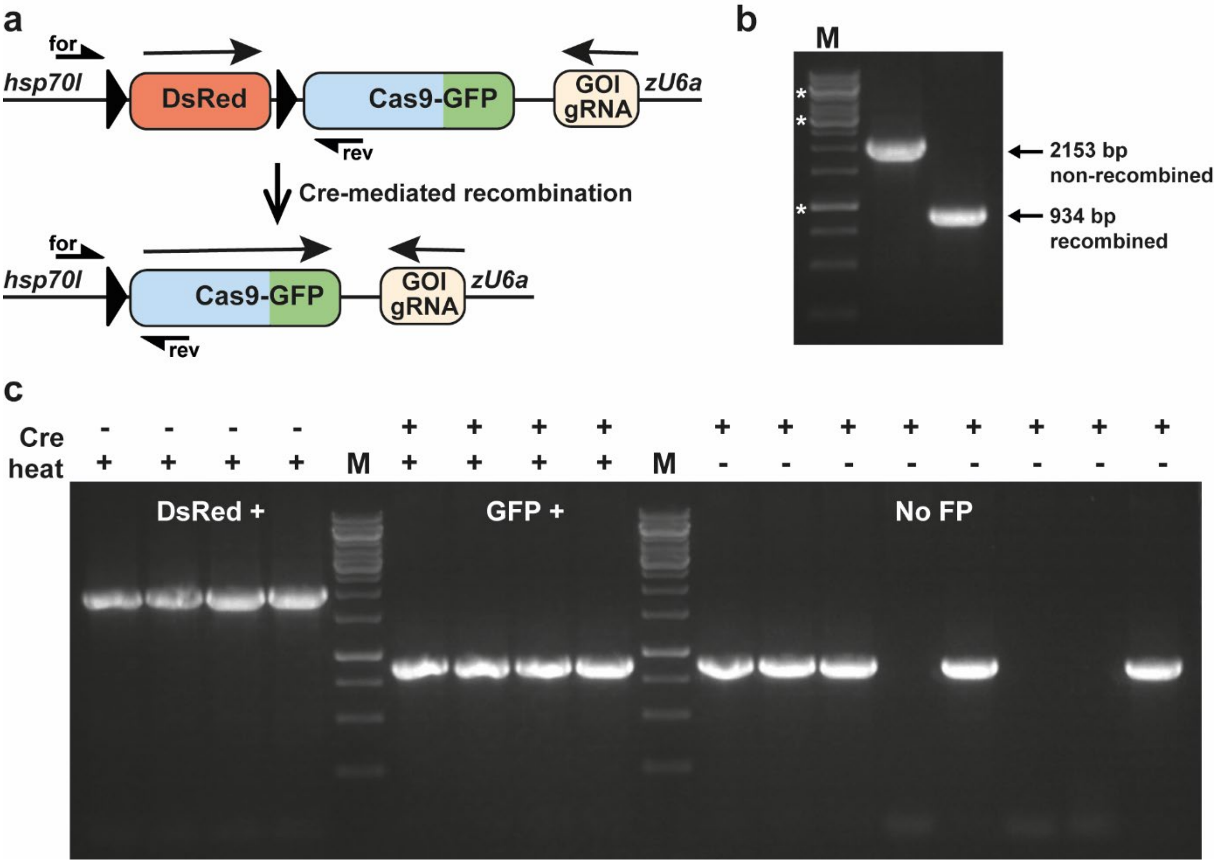

Supplementary figure 6: Genotyping of 3C *tyr* animals. **a,b** A PCR with a forward primer annealing in the *hsp70l* promoter and a reverse primer annealing in the 5' sequence of Cas9 allows the detection of the non-recombined (2153 bp) or recombined (934 bp) 3C construct. Primers are shown as half arrows. M: GeneRuler™ 1 kb DNA ladder marker for molecular size standard. Stronger reference bands (indicated with *) correspond to 1000, 3000 and 6000 bp. **c** Genotyping of 3C animals with (+) or without (-) Cre mRNA injection (Cre) or heat treatment (heat). DsRed+ and GFP+ indicates presence of the respective fluorescent protein prior to genomic DNA extraction. In the absence of a heat treatment no fluorescent protein (no FP) was observed. Lanes with no band represent wild-type siblings. Gel image shown is representative across >10 genotyping experiments showing the same result.

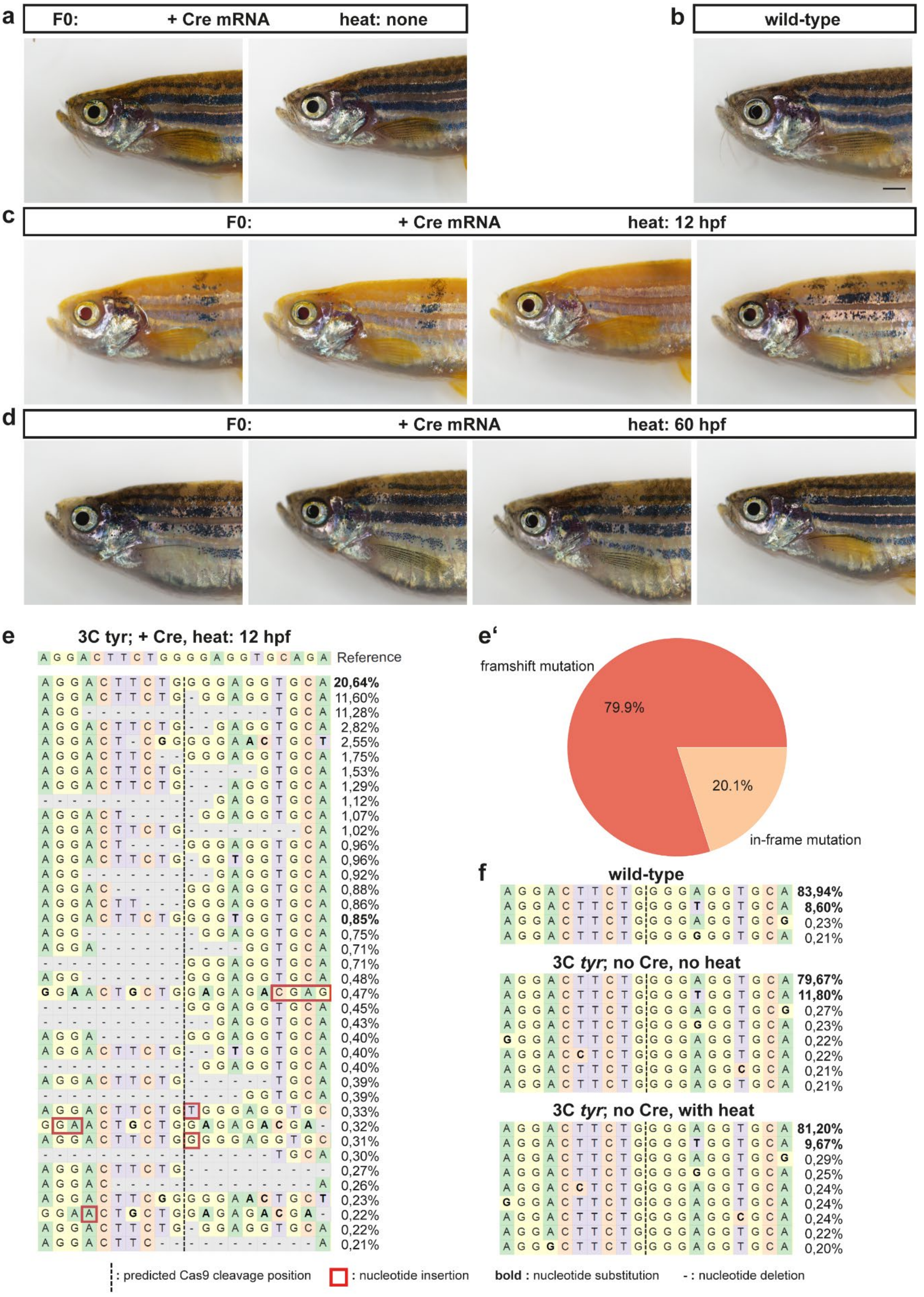

Supplementary figure 7: a,b Cre mRNA injected animals that were never subjected to a heat treatment do not show any pigmentation defects (a) and are indistinguishable from wild-type (b). **c,d** In contrast, variable pigmentation defects are observed in adult animals that were in-

jected with Cre mRNA at the 1-cell stage and heat treated at 12 hpf (c) and 60 hpf (d). Examples shown are representative of at least ten individual fish examined. Scale bar: 2 mm for A-D. **e,f** Allele frequency table for Cre mRNA injected 3C *tyr* animals heat treated at 12 hpf (e) and the three controls wild-type, 3C *tyr* without Cre mRNA injection and no heat treatment as well as 3C *tyr* without Cre mRNA injection but with a heat treatment (f). Nucleotides are indicated by unique colors (A = green; C = red; G = yellow; T = purple). Substitutions are shown in bold font. Red rectangles highlight inserted sequences. Horizontal dashed lines indicate deleted sequences. The vertical dashed line indicates the predicted cleavage site. **e'** Sequence analysis of Cre mRNA injected 3C *tyr* animals heat treated at 12 hpf shows that 79.1% and 20.1% of the introduced indels represent frameshift and in-frame mutations, respectively.
