# Supplementary material for: Cre-Controlled CRISPR mutagenesis provides fast and easy conditional gene inactivation in zebrafish
Source: Nat Commun. 2021 Feb 18;12:1125. doi: 10.1038/s41467-021-21427-6 (PMC7893016; doi:10.1038/s41467-021-21427-6)
Supplement: Supplementary file 2 — Reporting Summary [file 41467_2021_21427_MOESM2_ESM.pdf]

nature research

Corresponding author(s): Stefan Hans, Michael Brand

Last updated by author(s): Jan 22, 2021

Reporting Summary

Nature Research wishes to improve the reproducibility of the work that we publish. This form provides structure for consistency and transparency in reporting. For further information on Nature Research policies, see our Editorial Policies and the Editorial Policy Checklist.

Statistics

For all statistical analyses, confirm that the following items are present in the figure legend, table legend, main text, or Methods section.

n/a	Confirmed	
☐	☒	The exact sample size (n) for each experimental group/condition, given as a discrete number and unit of measurement
☐	☒	A statement on whether measurements were taken from distinct samples or whether the same sample was measured repeatedly
☐	☒	The statistical test(s) used AND whether they are one- or two-sided<br>*Only common tests should be described solely by name; describe more complex techniques in the Methods section.*
☒	☐	A description of all covariates tested
☒	☐	A description of any assumptions or corrections, such as tests of normality and adjustment for multiple comparisons
☐	☒	A full description of the statistical parameters including central tendency (e.g. means) or other basic estimates (e.g. regression coefficient) AND variation (e.g. standard deviation) or associated estimates of uncertainty (e.g. confidence intervals)
☐	☒	For null hypothesis testing, the test statistic (e.g. F, t, r) with confidence intervals, effect sizes, degrees of freedom and P value noted<br>*Give P values as exact values whenever suitable.*
☒	☐	For Bayesian analysis, information on the choice of priors and Markov chain Monte Carlo settings
☒	☐	For hierarchical and complex designs, identification of the appropriate level for tests and full reporting of outcomes
☒	☐	Estimates of effect sizes (e.g. Cohen's d, Pearson's r), indicating how they were calculated

Our web collection on statistics for biologists contains articles on many of the points above.

Software and code

Policy information about availability of computer code

Data collection	cellSens Dimension imaging software; Adobe Photoshop CC2015; Adobe Illustrator CC2015.
Data analysis	BD FACSDiva software; CRISPResso2 software; FIJI (ImageJ) software; PRISM7 (Graph-Pad) software.

For manuscripts utilizing custom algorithms or software that are central to the research but not yet described in published literature, software must be made available to editors and reviewers. We strongly encourage code deposition in a community repository (e.g. GitHub). See the Nature Research guidelines for submitting code & software for further information.

Data

Policy information about availability of data

All manuscripts must include a data availability statement. This statement should provide the following information, where applicable:

- Accession codes, unique identifiers, or web links for publicly available datasets
- A list of figures that have associated raw data
- A description of any restrictions on data availability

All sequencing data that support the findings of this study have been deposited in the NCBI Sequence Read Archive and are accessible through BioProject ID PRJNA693993. Source data are provided with this paper (Supplementary Tables 1&2).

Field-specific reporting

Life sciences study design

All studies must disclose on these points even when the disclosure is negative.

Sample size	Sample sizes were determined a priori according to a pilot experiment detecting effect sizes likely to be biologically relevant and limitations of obtaining and analyzing experimental material. Statistical analyses were performed only after all data had been collected. Formal power analyses were not performed.
Data exclusions	No data were excluded.
Replication	Analysis of pigmentation were replicated several times across all experimental groups. All attempts at replication were successfull.
Randomization	No formal method of randomization was used. Samples were collected randomly only based on the presence of the fluorescent proteins GFP or DsRed or the absence of both. Sample collection was always performed prior to the actual phenotypic analysis.
Blinding	Blinding was not possible because pigmentation defects are already visible.

Reporting for specific materials, systems and methods

We require information from authors about some types of materials, experimental systems and methods used in many studies. Here, indicate whether each material, system or method listed is relevant to your study. If you are not sure if a list item applies to your research, read the appropriate section before selecting a response.

Materials & experimental systems

n/a	Involved in the study	
☒	☐	Antibodies
☒	☐	Eukaryotic cell lines
☒	☐	Palaeontology and archaeology
☐	☒	Animals and other organisms
☒	☐	Human research participants
☒	☐	Clinical data
☒	☐	Dual use research of concern

Methods

n/a	Involved in the study	
☒	☐	ChIP-seq
☐	☒	Flow cytometry
☒	☐	MRI-based neuroimaging

Animals and other organisms

Policy information about studies involving animals; ARRIVE guidelines recommended for reporting animal research

Laboratory animals	Danio rerio, AB wild-type background
Wild animals	The study did not involve wild animals.
Field-collected samples	The study did not involve samples collected from the field.
Ethics oversight	Fish were kept according to FELASA guidelines (Aleström et al., 2020). All animal experiments were conducted according to the guidelines and under supervision of the Regierungspräsidium Dresden (permit: TVV 21/2018). All efforts were made to minimize animal suffering and the number of animals used.

Note that full information on the approval of the study protocol must also be provided in the manuscript.

Flow Cytometry

Plots

Confirm that:

☒ The axis labels state the marker and fluorochrome used (e.g. CD4-FITC).

☒ The axis scales are clearly visible. Include numbers along axes only for bottom left plot of group (a 'group' is an analysis of identical markers).

☒ All plots are contour plots with outliers or pseudocolor plots.

☒ A numerical value for number of cells or percentage (with statistics) is provided.

Methodology

Sample preparation	Tissue dissociation was conducted as described previously (Manoli and Driever, 2012). Briefly, embryos were removed from their chorions by pronase treatment (Westerfield, 2000), followed by deyolking at 4°C in 0.5% Ginzburg-Ringer without $CaCl_2$.

	Dissociation was conducted in trypsin-EDTA on ice. When embryos were completely dissociated, the reaction was stopped by adding Hi-FBS. The cells were pelleted, washed with PBS, resuspended in PBS and passed through a 40µM mesh filter prior to cell sorting.
Instrument	FACS was performed using an Aria II cell sorter (BD Biosciences).
Software	Flow cytometry data were analyzed using BD FACSDiva software.
Cell population abundance	The abundance of GFP-positive cells during sorting is shown in supplementary figure 2. No post-sorting was applied. Subsequent sequencing proved the enrichment of the sample.
Gating strategy	Forward and side scatter were used to gate for live, single cells, out of which GFP-positive cells were sorted and collected. For control cells from GFP-negative embryos the same gating strategy was employed.

☒ Tick this box to confirm that a figure exemplifying the gating strategy is provided in the Supplementary Information.
